# Topographic Analysis of the Nipple–Areolar Complex Sensation in Superomedial Pedicle Breast Reduction

**DOI:** 10.1007/s00266-024-04252-2

**Published:** 2024-07-31

**Authors:** Mehmet Sonmez, Murat Enes Saglam

**Affiliations:** 1 Department of Plastic, Reconstructive and Aesthetic Surgery, Ankara Yildirim Beyazit University, Universiteler Mah. 1604. Cad. No:9, Cankaya, Ankara, Turkey; 2 Plastic, Reconstructive and Aesthetic Surgery Clinic, Bursa City Hospital, Dogankoy Mahallesi, 16110 Nilufer, Bursa, Turkey

**Keywords:** Macromastia, Breast reduction, Superomedial pedicle, Nipple–Areolar complex, Innervation, Sensation

## Abstract

**Background:**

Surgeons meticulously perform breast reductions, while ensuring vascular integrity of the pedicle and Nipple–Areolar complex (NAC) to prevent any complication. It is crucial to remember that loss of sensation is also substantial complication, mainly due to unique characteristic features of the NAC. This study aimed to compare early and long-term sensory results by performing topographic analysis of NAC sensation after superomedial pedicle breast reduction.

**Methods:**

A prospective study was conducted by including nonrandomized female patients who underwent breast reduction surgery with wise pattern excision superomedial pedicle technique between January 2019 and June 2022. Semmes-Weinstein Monofilament (SWM) test performed at preoperatively, 3–6 months and 15–18 months postoperatively. NAC complex was divided into four equal quadrants and nipple: superomedial (SM), inferomedial (IM), inferolateral (IL), superolateral (SL) and Nipple (N). Touch-Test^®^ Sensory Evaluator Chart was used to evaluate sensory results.

**Results:**

None of the patients had any loss of sensation during preoperative SWM test. In postoperative 3–6 months, statistically significant differences were observed between N and SL (*p* = 0.002), SL and IM (*p* < 0.05), SM and IM (*p* < 0.05). In postoperative 15–18 months, there was no difference between the quadrants and nipple (*p* = 0.07). In early and long-term comparisons of the same quadrants, IL less pronounced than other quadrant comparisons (*p* = 0.034). A statistical difference was observed in overall NAC score (*p* < 0.05).

**Conclusions:**

It would be beneficial to inform patients overall NAC sensation in the postoperative may not be as good as preoperative, there might be variations in NAC sensation across different quadrants in early period.

**Level of Evidence IV:**

This journal requires that authors assign a level of evidence to each article. For a full description of these Evidence-Based Medicine ratings, please refer to the Table of Contents or the online Instructions to Authors www.springer.com/00266.

## Introduction

Breast reduction surgeries aim to remove excess breast tissue from breasts larger than the average size [1]. Surgeons carefully manipulate the remaining breast tissue to shape the breast in an appearance characterized by appropriate projection, a conical shape, and an overall aesthetic appeal. Pedicled breast reduction techniques are the preferred approach in cases without contraindications. These techniques yield superior aesthetic results and ensure the preservation of breastfeeding functionality [2, 3]. Surgeons meticulously carry out the operation, while ensuring the vascular integrity of the pedicle and the Nipple–Areolar Complex (NAC) to prevent any vascular complications. Nonetheless, it is crucial to remember that the loss of sensation is also a substantial complication due to the unique characteristic features of the NAC [4].

The superomedial pedicle technique has become popular since its definition due to several factors [5]. These include consistent anatomical properties, reliable outcomes and shorter operation time with less extensive de-epithelialization than alternative techniques [6]. Additionally, it reduces the risk of long-term bottoming-out deformity, ensuring more sustainable aesthetics [7]. It stands out with its unique ability to enhance upper pole fullness, distinguishing it from other approaches [8].

Blood supply to the superomedial pedicle arises from the second and third intercostal spaces, branches of the internal mammary artery extend into the pedicle [9]. These vessels are conveniently close to the skin surface, allowing for the desired pedicle thickness adjustment. Nipple sensation primarily relies on the lateral cutaneous branch of the fourth intercostal nerve in the natural unoperated breast anatomy [10]. All sides except the superomedial base are incised during the elevation of the superomedial pedicle, and the pedicle undergoes a 90° rotation as it is repositioned, causing a topographic shift of the NAC [11].

The NAC is a highly specialized anatomical region, recognized as an erogenous zone and highly responsive to stimulation [12]. Its sensory preservation is crucial, especially for patients undergoing breast surgery, as it can impact sexual satisfaction [13]. It is essential for breastfeeding patients, and preserving its sensation is vital for the milk ejection reflex and nurturing the mother-child bond [14, 15].

There was inadequate study in the literature that compares early and long-term results of NAC sensation after superomedial pedicled breast reduction surgeries, and the outcomes of these limited number of studies were contradictory. This study aimed to compare early and long-term sensory results by performing a topographic analysis of NAC sensation after superomedial pedicle breast reduction.

## Materials and Methods

### Patients

A prospective study was carried out by including non-randomized female patients who underwent breast reduction surgery with wise pattern excision superomedial pedicle technique in a single center between January 2019 and June 2022. Patient who had no previous breast surgery, known medical comorbidity, and smoking history enrolled in this study. According to the proposed classification of gigantomastia described by Dancey et al, our patient sample was consisted of Group 1b patients (BMI ≤ 30, Idiopathic, spontaneous condition of excessive breast growth) [16]. Informed consent was obtained from each patient, and the study received approval from the local ethics committee. The Semmes-Weinstein Monofilament (SWM) test was performed on each breast to confirm that there was not preoperatively sensory loss in the NAC. All surgical procedures were performed by the senior author.

### Preoperative Markings

**Fig. 1:**
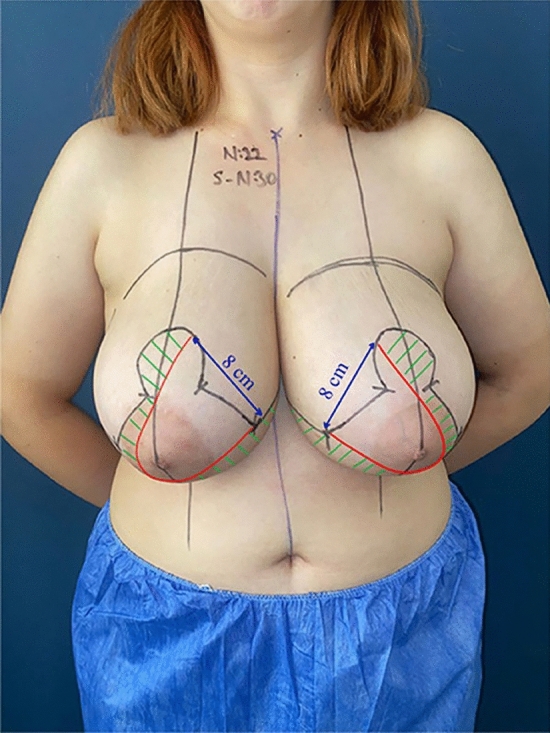
Preoperative markings of superomedial breast reduction technique. Sternal notch-nipple distance: 30 cm, new nipple position: 22 cm, the superomedial pedicle base: 8 cm

Markings were made when the patient was standing upright with arms at their sides. Sternal notch (SN) was marked. A line was drawn down the midline from the SN. A longitudinal tape measure was hung to draw the breast meridian for both sides, passing through the midclavicular line. The SN-Nipple distance was documented. The Pitanguy point was located on the breast meridian. It was ensured that the center line distances of these points determined on both sides were equal and ideally 10 to 12 cm. The Wise pattern was drawn 2 cm above the Pitanguy point, with legs extending 6 cm on both sides. The inframammary fold (IMF) was marked within 2 cm of the midline and to the anterior axillary line. Pattern legs were joined on both sides by the IMF line, and any asymmetries were corrected. Finally, the base of the superomedial pedicle was set at 8 cm (Fig. 1).

### Surgical Procedure

**Fig. 2:**
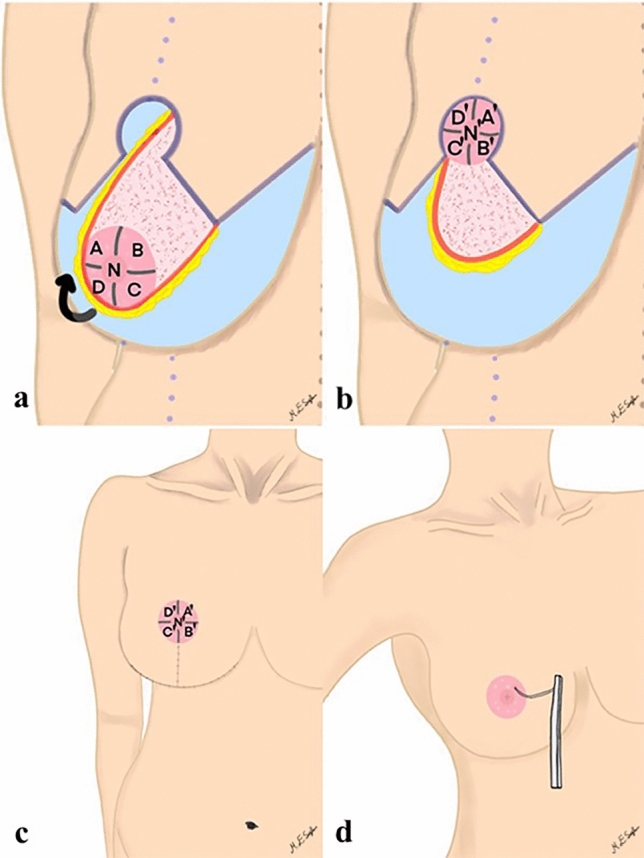
Illustration of surgical procedure on the right breast. **a** Elevation of superomedial pedicle and preoperative topographic position of Nipple–Areolar Complex; A: Superolateral, B: Superomedial, C: Inferomedial, D: Inferolateral, N: Nipple; blue area represents the tissue that has been excised; black arrow indicates the rotation direction. **b** Adaptation the Nipple–Areolar Complex by rotating 90° with the rotation of the pedicle, and postoperative topographic position of Nipple Areolar Complex; A': Superomedial, B': Inferomedial, C': Inferolateral, D': Superolateral, N': Nipple; blue area represents the tissue that has been excised. **c** Final appearance and position of the Nipple–Areolar Complex. **d** Application of Semmes-Weinstein Monofilament test, the examiner applies nylon filaments perpendicularly to the Nipple–Areolar Complex and allows the filaments to bend without exerting additional force

All patients were operated under general anesthesia. Tumescence solution was used in the incision lines and excision areas [17]. The new NAC was marked with a 40-mm-diameter cookie cutter. Pedicle de-epithelialization was performed. The superomedial pedicle was incised following the markings and released from the pectoral fascia sufficiently to rotate, and excisions were performed. The superomedial pedicle and accompanying NAC were rotated 90°, and the NAC was adapted to its new location (Fig. 2a and b). Hemovac drains were placed on each side. In subcutaneous dermal repair, 3-0 Vicryl^®^ (polyglactin 910) was used for facing the pillars in addition to non-NAC areas, and 4-0 Vicryl^®^ was used for NAC. The subcuticular technique was preferred for skin repair, while 4-0 Monocryl^®^ (poliglecaprone 25) was used in non-NAC areas, and 5-0 Monocryl^®^ was used for NAC.

### Evaluation

The SWM test, using The Touch-Test^®^ Sensory Evaluators (North Coast Medical, Inc, California, USA) kit, was conducted at 3–6 months and 15–18 months postoperatively to assess NAC sensation. Patients were seated on an examination table in a quiet room, with eyes closed and without clothing. The areolar tissue was divided into four quadrants (A', B', C', and D'), corresponding to superomedial (SM), inferomedial (IM), inferolateral (IL), and superolateral (SL) quadrants of the postoperative areola. The nipple (N) was the fifth region (Fig. 2c). Nylon filaments were applied to the NAC perpendicularly, and the examiner allowed the filaments to bend without exerting additional force (Fig. 2d). Patients were asked if they felt the stimulus and to specify the location. In cases where there was no sensation, the same size of monofilament test was repeated in the same region. A thicker filament was used still if there was no sensation. The Touch-Test^®^ Sensory Evaluator Chart was employed to evaluate results. Scores were assigned based on plantar threshold values: normal sensation (3 points), diminished light touch (2 points), diminished protective sensation (1 point), and loss of protective and deep pressure sensation (0 points) (Table 1). The scores for all quadrants and the nipple were summed to obtain the overall NAC result. Scores <5 were considered poor, 5–10 as fair, and >10 as good.

**Table 1:** Touch-Test^®^ sensory evaluator chart and points

Evaluator size	Target force^*^ in grams	Plantar thresholds	Points
1.65–3.61	0.008–0.400	Normal	3
3.84–4.31	0.600–2.000	Diminished Light Touch	2
4.56–4.93	4.000–8.000	Diminished Protective Sensation	1
5.07–6.65	10.000–300.000	Loss of Protective Sensation Deep Pressure Sensation	0

*Individually calibrated within a 5% standard deviation

### Statistical Analysis

The scores of all quadrants and nipple within the same period were statistically compared to each other. Early (3–6 months) and long-term results (15–18 months) of the same quadrants and nipple were separately compared. Overall NAC scores were analyzed to determine whether there was a difference between the early and long-term results. Pearson correlation analysis was performed to determine the correlation between amounts of breast tissue removed and long-term overall NAC sensation scores, and the t-test performed to determine the difference between preoperative breast ptosis degrees and long-term overall NAC sensation scores. *p* < 0.05 was considered as statistically significant. (SPSS software ver.25, IBM^®^, New York, USA)

## Results

The study involved 16 female patients who had undergone breast reduction surgery with superomedial pedicle technique, and a total of 32 breasts were examined, consisting of 16 on the right and 16 on the left. The mean age of the patients was 32.1 ± 6.8 years. Mean body mass index (BMI) was 24.4 ± 2.2 kg/m^2^.

**Fig. 3:**
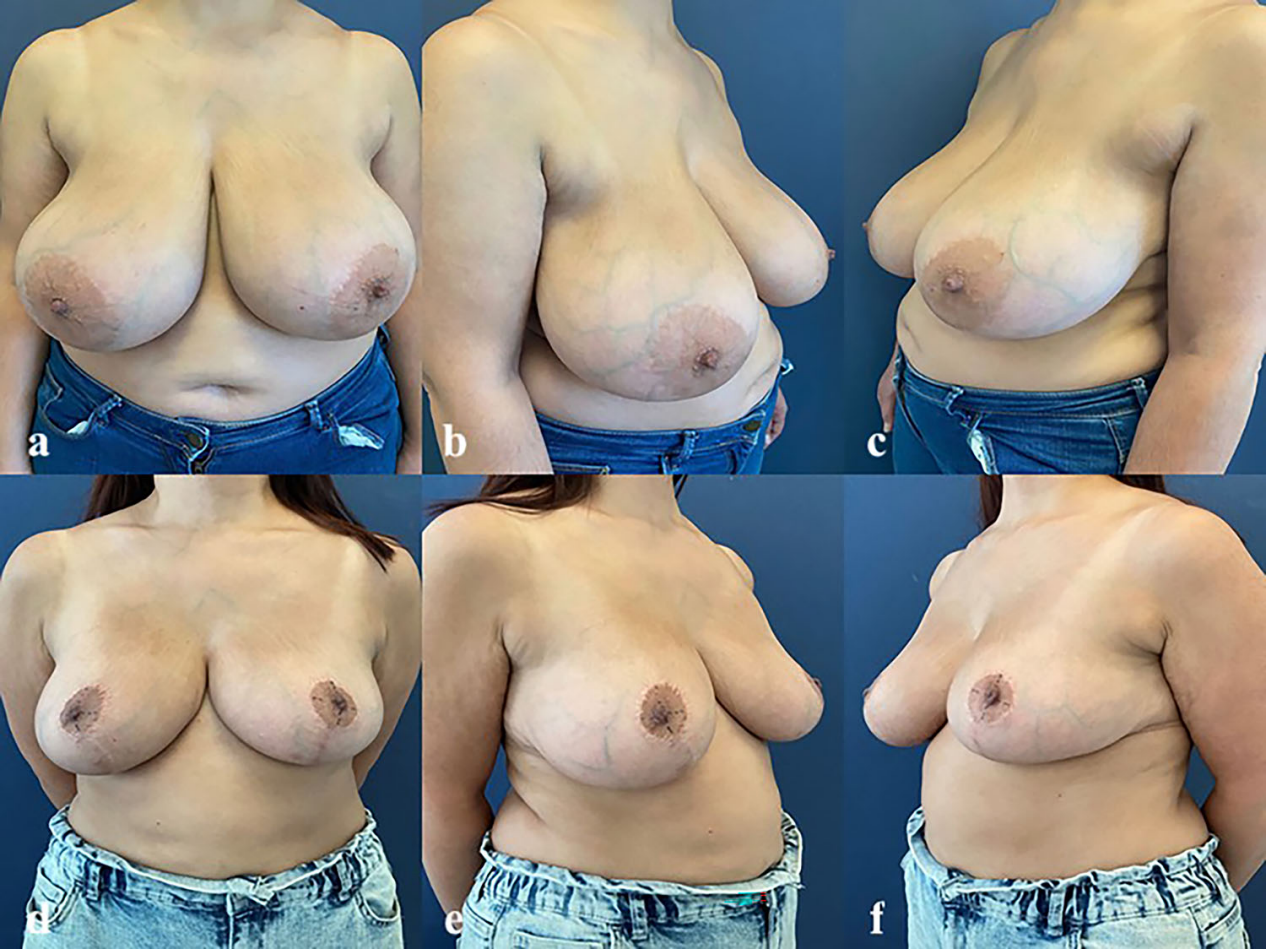
Pre- and post – operative views of a 29-year-old female patient. Sternal notch – nipple distance: 31 cm, new nipple position: 22 cm, amount of tissue excised: 675 g/641 g (right/left). **a–c** Preoperative views. **d–f** Postoperative 12 month views

**Fig. 4:**
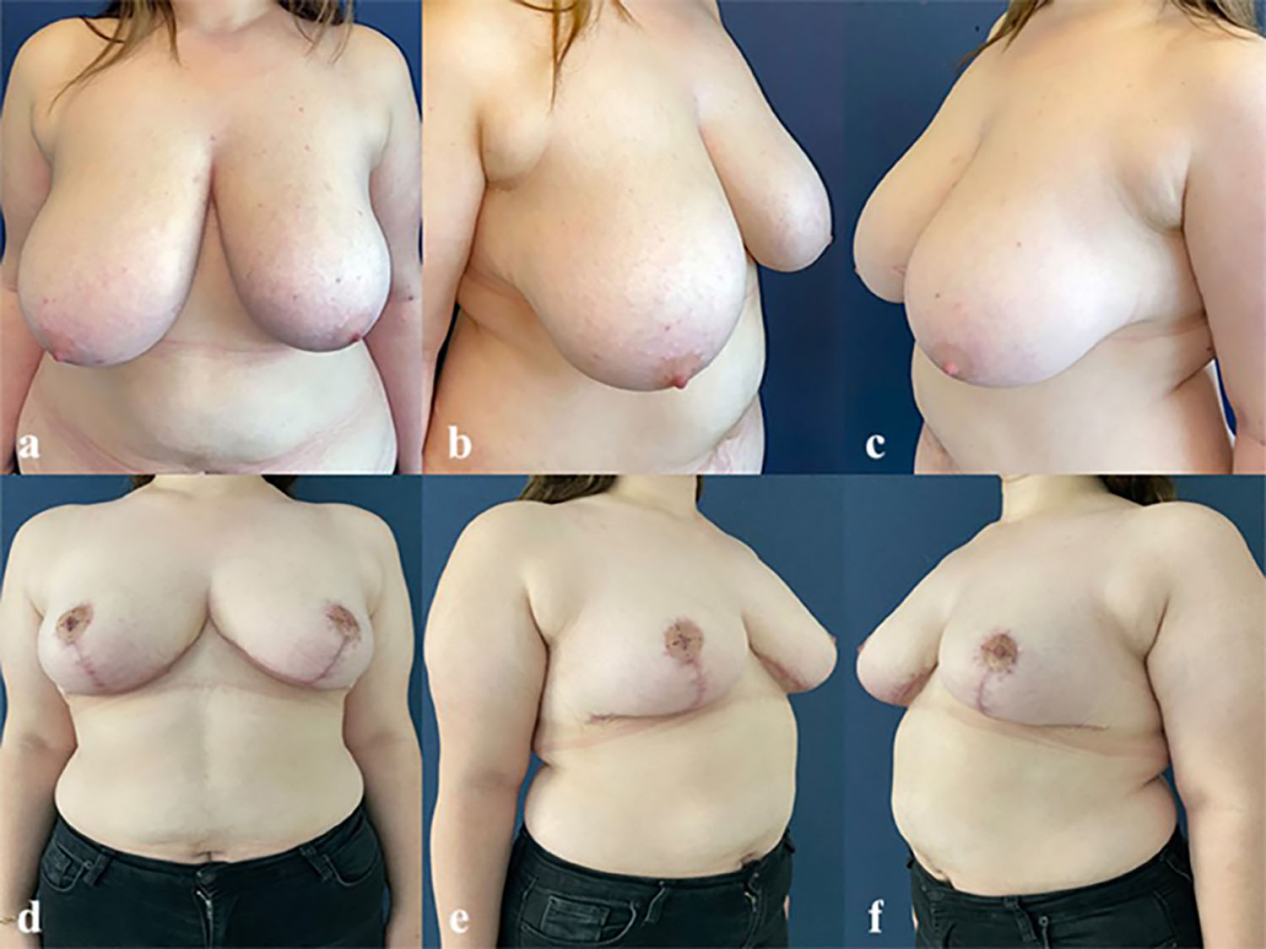
Pre-and post- operative views of a 41-year-old female patient. Sternal notch-nipple distance: 33 cm, new nipple position: 23 cm, amount of tissue excised: 806 g/770 g (right/left). **a–c** Preoperative views. **d–f** Postoperative 18 month views

None of the patients reported any loss of sensation during the preoperative SWM test, and the overall NAC score was 15 points. Mean preoperative SN-Nipple distance was 30.4 ± 2.0 cm. Postoperative SN-Nipple distance was 21.9 ± 1.4 cm. The amount of tissue removed after reduction was 563.1 ± 150.3 g. Among the breasts that underwent superomedial breast reduction surgery, fifteen (47%) were grade 2 and seventeen (53%) were grade 3 according to Regnault classification [18]. Patient characteristics and measurements can be shown in Table 2. Figures 3 and 4 shows preoperative and postoperative photographs.

**Table 2:** Patient characteristics and measurements

	Mean	Sd	Min	Max
Age (years)	32.1	6.8	19	41
BMI (kg/m^2^)	24.4	2.2	20.6	28.9
Preoperative SN-Nipple distance (cm)	30.4	2.0	27.5	34.0
Postoperative SN-Nipple distance (cm)	21.9	1.4	20.0	25.0
Reduction weight per breast (g)	563.1	150.3	364.0	860.0

*Sd* Standard deviation, *Min* minimum, *Max* maximum, *BMI* body mass index, *kg* kilograms, *m* meters, *SN* sternal notch, *g* grams

During the early postoperative period, one breast developed a hematoma, which was drained in the operating room six hours after surgery. There was no loss of NAC in any patient, and no major complications occurred. Additionally, seven breasts showed minor necrosis and dehiscence at the T junction area, effectively managed through conservative secondary wound healing with dressings.

The topographic sensory results of the NAC were found to deviate from a normal distribution (*p* < 0.05) during the postoperative 3–6 months (average 4.9), which corresponds to the early period following superomedial pedicled breast reduction surgery. Kruskal-Wallis test performed to determine the difference between the quadrants and nipple, revealed that there was a difference between the groups (*p* < 0.05) (Table 3). The quadrants and nipple were compared in pairs using the Mann-Whitney U test with Bonferroni correction to determine which were different. Statistically significant differences were observed between the N and SL (*p* = 0.002), the SL and IM (*p* < 0.05), and the SM and IM (*p* < 0.05). Overall mean NAC score was 5.9, which corresponds to fair result.

**Table 3:** Postoperative 3–6 months points of NAC sensation

	Mean	Sd	Min	Max	Kruskal–Wallis test	
					*χ*^2^	*p* value
N	1.5	0.6	0	2.0	28.5	<0.05
SL	0.6	0.7	0	2.0		
SM	0.8	0.6	0	2.0		
IM	1.7	0.5	1.0	2.0		
IL	1.3	0.9	0	2.0		
O	5.9	2.2	2.0	9.0		

*NAC* Nipple–Areolar Complex, *Sd* standard deviation, *Min* minimum, *Max* maximum, *N* nipple, *SL* supero lateral, quadrant *SM* supero medial quadrant, *IM* infero medial quadrant, *IL* infero lateral quadrant, *O* overall

In the long-term postoperative period, at 15–18 months, (average 16.3) it was noted that the quadrants and nipple did not exhibit a normal distribution according to the scoring performed for the topographic SWM test results (*p* < 0.05). Kruskal-Wallis test performed to determine the difference between the quadrants and nipple showed that there was no difference between the groups (*p* = 0.07) (Table 4). Overall mean score was 10.8 which corresponds to good result.

**Table 4:** Postoperative 15–18 months points of NAC sensation

	Mean	Sd	Min	Max	Kruskal–Wallis test	
					*χ*^2^	*p* value
N	2.4	0.7	1.0	3.0	8.7	0.07
SL	1.9	0.7	1.0	3.0		
SM	1.9	0.9	0	3.0		
IM	2.6	0.6	1.0	3.0		
IL	2.0	0.9	0	3.0		
O	10.8	3.1	4.0	15.0		

*NAC* Nipple–Areolar Complex, *Sd* standard deviation, *Min* minimum, *Max* maximum, *N* nipple, *SL* supero lateral quadrant, *SM* supero medial quadrant, *IM* infero medial quadrant, *IL* infero lateral quadrant, *O* overall

In the early and long-term comparisons of the same regions, a statistical difference was observed between 3–6 months and 15–18 months postoperatively for N, SL, SM, and IM (*p* < 0.05). In the comparison of the IL quadrant, a statistically significant difference was noted between the early and long-term periods, although it was less pronounced than in other quadrant comparisons (*p* = 0.034). A statistical difference was also observed in the overall NAC score (*p* < 0.05) (Table 5).

**Table 5:** Comparison of postoperative 3–6 months points and 15–18 months points

	*p* value
N Postoperative 3–6 months and 15–18 months	0.002
SL Postoperative 3–6 months and 15–18 months	< 0.05
SM Postoperative 3–6 months and 15–18 months	0.001
IM Postoperative 3–6 months and 15–18 months	0.034
IL Postoperative 3–6 months and 15–18 months	< 0.05
O Postoperative 3–6 months and 15–18 months	< 0.05

*N* Nipple, *SL* supero lateral quadrant, *SM* supero medial quadrant, *IM* infero medial quadrant, *IL* infero lateral quadrant, *O* overall

A moderate negative correlation was observed between the amount of breast tissue removed and long-term overall NAC sensation score (*r* = −0.42). There was no statistically significant difference in the long-term overall NAC sensation scores of patients with grade 2 and grade 3 ptosis (*p* = 0.08).

## Discussion

Maintaining the sensation of the NAC is equally vital as achieving a favorable cosmetic outcome in breast reduction surgeries. In an anatomical study by Craig and Sykes, they discovered that the NAC was consistently supplied with nerve branches originating from the anterior cutaneous nerves of the third, fourth, and fifth intercostal nerves, as well as branches from the lateral cutaneous nerves of the fourth and fifth intercostal nerves [19]. Wuringer et al. reported that, in most cadavers, the deep branch of the lateral branch of the fourth intercostal nerve, and less frequently the fifth intercostal nerve, runs adjacent to a horizontal fibrous septum, providing innervation to the nipple [20]. Likewise, there is a study in which the terminal branches of the fourth and fifth intercostal nerves become superficial, forming a plexus just below the dermal layer near the NAC [21]. According to the literature, multiple nerves within the unoperated breast tissue, along with their branches, play a crucial role in the sensory function of the NAC [10].

Various techniques can be used when performing breast reduction. When a free nipple graft is used, the NAC has as much sensation as it is reinnervated as a result of graft healing in the new site, and this has the worst outcomes compared to other technique outcomes [22]. In pedicled breast reduction surgeries, it is unavoidable that some of the nerves innervating the NAC may be severed during the elevation of the pedicle and excision of the other areas. However, preserving certain cutaneous nerves depends on the specific type of pedicle used. Preserving sensation is not only about avoiding nerve severance during breast reduction surgery; even minor traction or compression can potentially damage these nerves, leading to a loss of function. This functional impairment, depending on the grade of the damage, can either be reversible or irreversible [23].

When the superomedial pedicle breast reduction technique was first described, the pedicle and NAC were transposed to the new location as a dermal flap. Even when transposed in this manner, Orlando et al. reported improved sensory return in their study compared to the superior pedicle approach, although specific measurements were not mentioned [5]. Various modifications were introduced, including the rotation and repositioning of the pedicle and NAC. This emphasizes the preservation of sensation [24]. The implementation of the superomedial pedicle technique by Hall-Findlay, which incorporates a vertical incision with a short scar, has contributed to the increased popularity of this pedicle [25].

Literature on NAC sensation mainly compared different techniques. These studies typically had relatively short follow-up periods, considering the time required for nerve healing and regeneration [26–29]. Moreover, it is worth noting that many of these studies lack preoperative NAC sensory assessments. In a study comparing the superior and inferior pedicle with SMW test results at postoperative six months, the inferior pedicle was found to be slightly better in nipple sensation [30]. In another anatomical and histological comparison of the same pedicles conducted by the previous author, the conclusion was drawn that nerve damage could occur during pedicle dissection, thinning, plication, or suturing, regardless of the pedicle used [31]. In another research comparing the inferior and medial pedicle with the Pressure Specified Sensory Device^®^ (PSSD) test, no statistically significant difference was found between both techniques [32]. In a different study comparing the superomedial and superior pedicle with the monofilament test, it was indicated that the NAC sensation of the superomedial pedicle was statistically superior. However, even though the nerve fibers entering the superomedial pedicle were better preserved compared to the superior pedicle, the study concluded that this difference was not clinically significant [33]. Watfa et al. compared the superomedial and inferior pedicle with the SWM test and found that there were slight differences in the long-term, but the superomedial pedicle exhibited advantages in terms of NAC sensation [34]. Payton et al. assessed NAC sensation by running a finger across the NAC both before and after superomedial pedicle breast reduction surgery, and they reported a decrease in sensation after the surgery [28].

In the current study, the research findings indicated that NAC sensation decreased in the early postoperative period (3–6 months) compared to the preoperative sensation. However, over time (15–18 months) following breast reduction surgeries performed with the superomedial technique, there was an improvement in NAC sensation compared to the early results. Similarly, Ferreira et al. and Garcia et al. mentioned that there was a decrease in NAC sensation at six months after superomedial pedicle breast reduction surgery compared to the preoperative period and that the follow-up period was short as a study limitation [35, 36]. Despite the decrease in sensation observed in Garcia et al.'s study, it was determined that this reduction in sensation did not have a significant impact on the sexual function of the patients, as indicated by the results of the Female Sexual Function Index [37]. In the current study, patients who did not regain their preoperative sensation did not report any complaints in this regard.

In the results of our study, it was observed that only 50% of the breasts could reach the preoperative score evaluated as good for NAC sensation in the superomedial breast reduction technique in 15–18 months postoperatively, and the result was fair in 46% of breasts. A NAC with a poor score, even in the long-term was a breast developed postoperative hematoma. When the literature was examined, no study on the long-term sensory results of breasts with hematoma was found. In contrast to our results, in a study by Muslu et al. using a digital algometer, it was found that NAC returned to preoperative sensation at six months after breast reduction surgeries using superomedial pedicle and inferior pedicle [26]. In another study using the inferior pedicle technique, it was reported that sensory return was observed in 90.5% of patients. However, this study did not specify the postoperative time at which the sensory return occurred, and it did not provide any classification regarding the sensory outcomes [37]. The results of this topographic study revealed that the most favorable sensory outcomes in the early period (3–6 months) were observed in the N region and in the most proximal quadrant to the preoperative base of the superomedial pedicle IM. The least favorable early outcomes were observed in the quadrant corresponding to the preoperative most distal part SL, similar to the findings in a study by Hamdi et al., which compared the superior and inferior pedicle techniques [30]. When comparing the early and long-term results within the same quadrants, it was observed that all quadrants showed a statistically significant difference. However, the difference in the IL region was weaker when compared to the differences observed in comparisons involving other quadrants. This result is thought to be associated with the fact that the IL quadrant undergoes the most twisting during pedicle rotation, which may result in comparatively less effective nerve healing. While, the complete physiology of innervation restoration following reduction surgery has not been fully elucidated, Hamdi et al. proposed that severed cutaneous nerve branches or the persistence of cutaneous innervation contribute to restoring NAC sensation [31]. Our results align with this perspective and demonstrate that recovery levels of topographic regions also vary over time. In addition, we found a moderate negative correlation between the amount of breast tissue removed and long-term NAC sensation, and this result was contrary to the literature [13, 38, 39].

Our study stands out from other studies due to the composition of the patient group, which consists of individuals in their reproductive years with no known diseases, similar breast sizes, and all patients were operated on by a single surgeon. Additionally, smoking is a known factor that can have a negative impact on nerve recovery [40]. The findings of the current study were not influenced by patients who smoke because there were no smokers in the study. Sensory examination was conducted using the SWM test, and comparisons were made and scored based on plantar threshold levels. The selection of plantar threshold levels was influenced by the fact that the sensation of the NAC, recognized as one of the erogenous regions in the somatosensory cortex, is represented in an area closely associated with foot sensation [41].

This study has some limitations. First, this study is a single center study. Second, sample size is relatively small. Third, our sample was consisted of only the patients that have a ≤30 BMI score and idiopathic gigantomastia. Therefore, a multicentered study with larger sample size and on different gigantomastia groups could be conducted in the future to obtain more data in this field.

## Conclusions

The NAC sensation is affected during breast reduction surgery using the superomedial pedicle, and there is not enough sensory return in the early period (3–6 months), however, sensory return in the long-term (15–18 months) is the same as before surgery in half of the breasts. On the other hand, nearly half of the patients’ NAC sensation could not reach to preoperative sensation status after superomedial breast reduction in long-term. Therefore, it would be beneficial to inform patients who have undergone the superomedial pedicle breast reduction technique that the overall NAC sensation in the postoperative breasts may not be as good as preoperative, and there might also be variations in NAC sensation across different regions in the early postoperative period.
